# Delayed repair of radiation induced clustered DNA damage: Friend or foe?

**DOI:** 10.1016/j.mrfmmm.2010.11.003

**Published:** 2011-06-03

**Authors:** Laura J. Eccles, Peter O’Neill, Martine E. Lomax

**Affiliations:** DNA Damage Group, Gray Institute for Radiation Oncology and Biology, University of Oxford, Old Road Campus Research Building, Roosevelt Drive, Oxford OX3 7DQ, UK

**Keywords:** AP, abasic, DSB, double strand breaks, SSB, single strand breaks, LET, linear energy transfer, BER, base excision repair, 8-oxoG, 8-oxo-7,8-dihydroguanine, Tg, thymine glycol, DHT, 5,6-dihydrothymine, hU, 5-hydroxyuracil, 8-oxoA, 8-oxo-7,8-dihydroadenine, DHU, dihydrouracil, Non-DSB clusters, Ionizing radiation, Base excision repair, Mutation induction

## Abstract

A signature of ionizing radiation exposure is the induction of DNA clustered damaged sites, defined as two or more lesions within one to two helical turns of DNA by passage of a single radiation track. Clustered damage is made up of double strand breaks (DSB) with associated base lesions or abasic (AP) sites, and non-DSB clusters comprised of base lesions, AP sites and single strand breaks. This review will concentrate on the experimental findings of the processing of non-DSB clustered damaged sites. It has been shown that non-DSB clustered damaged sites compromise the base excision repair pathway leading to the lifetime extension of the lesions within the cluster, compared to isolated lesions, thus the likelihood that the lesions persist to replication and induce mutation is increased. In addition certain non-DSB clustered damaged sites are processed within the cell to form additional DSB. The use of *E. coli* to demonstrate that clustering of DNA lesions is the major cause of the detrimental consequences of ionizing radiation is also discussed. The delayed repair of non-DSB clustered damaged sites in humans can be seen as a “friend”, leading to cell killing in tumour cells or as a “foe”, resulting in the formation of mutations and genetic instability in normal tissue.

## 1 Radiation and clustered damage

**Fig. 1:**
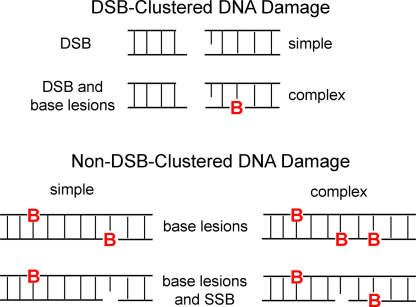
Schematic showing DSB and non-DSB clustered DNA damage. DSB can be simple or more complex with associated base lesions and AP sites. Non-DSB clustered damaged, defined as two or more lesions within one or two helical turns of DNA by passage of a single radiation track, increases in complexity with increasing LET of radiation. B, base lesion.

Reactive oxygen species produced during normal aerobic metabolism may interact with DNA to give a variety of types of lesion, many of which are chemically indistinguishable from those induced in cells by ionizing radiation [1,2]. However, the harmful effects of ionizing radiation to man have been proposed to arise largely from the formation of sub-classes of DNA damage called clustered DNA damage, or multiply damaged sites, which also include DNA double strand breaks (DSB) (Fig. 1). Clustered DNA damage sites, defined as two or more lesions formed within one or two helical turns of the DNA by passage of a single radiation track [3,4], are regarded as a signature of ionizing radiation exposure particularly as the likelihood of clustered damage sites arising endogenously is low as recently confirmed [5,6]. An important feature of ionizing radiation is the spatial distribution of DNA lesions induced as a consequence of energy being deposited unevenly along its track [7,8]. Sparsely ionizing radiation deposits most of its energy as a single ionisation event, but ∼30% of the ionisation events are formed closely together leading to the production of clusters of lesions whereas, densely ionizing radiation, such as α particles, produce an even higher density of DNA lesions within 10–20 base pair separation. Clustered DNA damage can be either bistranded, where the lesions are on opposing DNA strands, or tandem, where the lesions are on the same DNA strand.

Predictions from biophysical models of interactions of radiation tracks with DNA confirm that the complexity of the clusters increases with increasing ionization density of the radiation [3,9,10]. A percentage of DSB, defined as complex DSB, has associated additional lesion(s), such as single strand breaks (SSB) or base damage, in close proximity to the break termini [10,11]. Previous modelling studies [12] have shown that about 30–40% of low LET-induced DSB are complex with the yield increasing to >90% for high LET radiation [13]. Consistent with this prediction is the increased biological effects seen such as mutagenesis, carcinogenesis, lethality and the reduced reparability of DSB, with increasing ionization density of the radiation [1,2] thereby distinguishing radiation-induced clusters from readily repairable endogenous damage. The potential importance of clustered DNA damage was further highlighted from considerations that ∼10^3^ SSB induced by a dose of one Gy of radiation is equivalent to ∼2.6 million SSB being induced by hydrogen peroxide and resulting in a similar level of cellular inactivation [14]. It is the ability of ionizing radiation to produce clustered DNA damage sites, including DSB, against a background of endogenous damage, estimated to be induced at a rate of 10,000 DNA damages/cell/day [15], which leads to the biological effects of ionizing radiation. It is to be noted that the accurate measurement of endogenous damage is extremely difficult. Recent estimates of the background levels of 8-oxoG established by the European Standards Committee on Oxidative DNA Damage are 0.3–4.2 8-oxoG per 10^6^ guanine residues, orders of magnitude lower than previous estimates [16,17].

The induction of radiation-induced non-DSB clustered DNA damage sites in mammalian cells [18–21] and *E. coli* [22] has been confirmed experimentally, with a lower estimate of the yield of non-DSB clustered DNA damage being ∼4–8 times greater than that of prompt DSB. More recently it was shown the lifetime of non-DSB clustered damage induced in mammalian cells by radiation is considerably longer than that for isolated lesions [23,24], a finding that is consistent with the premise that mammalian cells have developed pathways to repair DNA damage [75] to minimise the biological consequences of endogenous DNA damage.

In this review we will concentrate on non-DSB clustered damage and present the experimental findings supporting the hypothesis that radiation-induced non-DSB clustered DNA damage sites are less repairable than isolated single lesions, e.g. those caused by aerobic metabolism [3,7,25,26] and as a consequence are either highly mutagenic or cytotoxic. Thus clustered DNA damage in human cells can be seen as a ‘foe’ if mutations are induced in a normal cell or as a ‘friend’ if they lead to tumour cell killing.

## 2 *In vitro* studies of the repair of clustered DNA damaged sites

As the chemical nature of lesions within ionizing radiation-induced clustered DNA damaged sites is the same as those that are formed endogenously in isolation the main pathway for the repair of DNA clustered damaged sites is the base excision repair (BER) pathway. In recent years a number of studies have been undertaken to investigate how efficiently lesions within clustered damaged sites are repaired, in comparison to isolated DNA lesions. As ionization radiation induced damage is a random event it is difficult to investigate in cells specific clusters, therefore short synthetic oligonucleotides have been designed with known DNA lesions, including mutagenic, non-mutagenic and DNA polymerase blocking lesions, placed at precise positions. Repair of these substrates has been carried out using either mammalian nuclear or whole cell extracts or by reconstituting the BER pathway with purified BER proteins. It is now well established that repair of lesions within a clustered DNA damage site is compromised compared to the repair of the same lesions present in isolation and the extent of retardation is dependent on the lesions within the cluster, the interlesion distance and the orientation of the lesions to each other. To clarify where in a cluster a lesion is positioned the following nomenclature will be used throughout this review. The lesions are said to be in the positive orientation when the opposing strand lesion is 3′ to the base opposite the reference lesion and to be in the negative orientation when the opposing strand lesion is 5′ to the base opposite the reference lesion. Likewise, with tandem lesions (those on the same DNA strand) the lesions are in the positive orientation when the second lesion is 3′ to the reference lesion and in the negative orientation when the second lesion is 5′ to the reference lesion. The number denotes the number of bases the second lesion is away from the reference lesion.

Using mammalian nuclear or whole cell extracts the repair of an abasic (AP) site or a SSB is reduced when a base lesion is within five bases of the AP site/SSB compared to the efficiency of repair of an AP site/SSB present in isolation. The nature of the base lesion was found to influence the extent of the reduction in repair. An 8-oxo-7,8-dihydroguanine (8-oxoG) lesion results in a 2–8 fold reduction in the efficiency of the repair of an opposing SSB and the effect was seen in both the positive and the negative orientations [27]. A similar effect was seen with thymine glycol (Tg), resulting in a 2.5 fold reduction [28,29] and with DHT a more modest reduction in the efficiency of SSB rejoining was observed of 1.4–2 fold, again over five bases in both orientations [30]. However when Tg was in the +1 position no repair of the SSB was seen as the Tg residue causes a block to repair synthesis by polymerase [29]. 8-OxoG and 5,6-dihydrothymine (DHT) reduce the efficiency of repair of a preformed SSB to a greater degree than an AP site. Not only is the degree of retardation in rejoining efficiency of the incised AP site lower, typically 1.3–2 fold, the distance over which the base lesion influences rejoining of the incised AP site is smaller, up to 5 bases in the negative orientation but only 1 base in the positive orientation [30,31]. The first step of repair of an AP site is its incision to give a SSB. At present this phenomenon has not been clearly explained but is thought to be due to the proteins recruited to repair the two different lesions. Indeed XRCC1 was found to play a role in the repair of AP sites within a clustered damaged site with a base lesion, but XRCC1 does not play a role in the repair of SSB in similar DNA clustered damaged sites [30,32].

In general the closer the base lesion is to the AP site/SSB the greater the reduction in the efficiency of repair of the AP site/SSB compared with an AP site/SSB when present as a solitary lesion. For example a Tg residue retards the rejoining of an incised AP site at 15 min by 2.3 fold when at position −1 but only by 1.4 fold when in position −5 [29], and 8-oxoG impairs the initial rate of rejoining of a SSB by 3.5 fold when positioned at +1 and by 2 fold when at +5 [27]. It was found that when a base lesion and an AP site/SSB are in the negative orientation the repair of the AP site/SSB proceeds by both long and short patch BER processes, whereas repair is primarily via the short patch BER pathway when the lesions are in the positive orientation [27,29–31]. The asymmetric use of the LP-BER pathway is not fully understood but may be due to local distortion of the DNA caused by the presence of more than one DNA lesion combined with the polymerase reading towards the base lesion when the AP site/SSB is in the negative orientation, whereas the polymerase reads away from the base lesion when the AP site/SSB is in the positive orientation. Repair by long and short patch BER is less efficient than that by short patch alone thus a base lesion in the negative orientation reduces the efficiency of repair of an AP site/SSB to a greater extent than a base lesion in a positive orientation [27,30,31]. The most striking example of this is when an 8-oxoG lesion is placed opposite a SSB. The initial rate of rejoining of the SSB is reduced by 8 fold when 8-oxoG is in the −5 position compared with 2.5 fold at −1, 3.5 fold at +1 and 2 fold at +5. However this difference is less pronounced at 60 min with reductions in the efficiency of rejoining of the SSB of 5.3 fold when 8-oxoG is at −5, 2.7 fold at −1, 2.3 fold at +1 and 1.6 fold at +5 [27].

Using purified BER proteins it is possible to identify which proteins in the repair pathway are most affected by the presence of another lesion in close proximity. A large number of studies have identified that the initial stage of the BER pathway, namely the rate of removal of the damaged base by DNA glycosylases, is compromised when another DNA lesion is in close proximity. This is thought to be due mainly to the presence of a second lesion within the footprint of the glycosylase so that the glycosylase cannot properly bind to the base lesion. In general terms an AP site or a SSB may strongly impair the rate of excision of a base lesion by purified glycosylases when located up to five bases from the base lesion. The extent of 8-oxoG excision by Fpg, yOGG1, hOGG1 and xrs5 nuclear extracts is reduced by up to 10 fold by an opposing AP site and by SSB with various termini, created by the action of different BER proteins. With a SSB with 3′ and 5′ phosphate termini the reduction is 15–30 fold when the SSB is one base from the 8-oxoG lesion in either orientation [33–36]. Similarly the efficiency of excision of Tg by Nth and DHT by Nth, Fpg and xrs5 nuclear extracts is markedly reduced by an opposing AP site, by 20–50 fold when the two lesions are in certain positions to each other [29,37]. Conversely a base lesion on the opposing DNA strand does not impair the incision of an AP site. Indeed studies to date have not found AP endonucleases present in nuclear or whole cell extracts to be influenced by a nearby base lesion (not including AP sites) on the opposing DNA strand when incising an AP site [28,31,37,38]. In addition purified APE1, Nth and Fpg are not retarded by opposing 8-oxoG, DHT or 8-oxoA lesions in their ability to incise an AP site [36,37]. However, AP site incision by yOGG1 is impaired by an opposing 8-oxoG [34] and an opposing Tg impairs AP site incision by the *E. coli* proteins Fpg, Nth and ExoIII [29].

In bistranded clustered DNA damaged sites comprised of either an AP site or a SSB and 8-oxoG impairment of polymerase β repair synthesis was not seen [27,31,38], however, bistranded clusters comprised of an AP site/SSB and Tg/DHT do reduce the efficiency of nucleotide incorporation by polymerase β at certain positions. Tg is a known block to polymerase [39] and totally blocks repair synthesis of an AP site/SSB one base in the positive orientation [29]. When the Tg residue is directly opposite an AP site polymerase β incorporation is reduced by 3 fold [28]. Similarly, DHT confers a slight retardation on repair synthesis by polymerase β of 1.3 fold, when an AP site is in the +1 and −1 positions [30]. Polymerase β also has a dRPase activity to remove the sugar residue following AP endonuclease cleavage. This activity of polymerase β has been shown not to be affected by another lesion in close proximity [28,30,32]. In addition strand displacement synthesis by both polymerase β and polymerase δ and flap cleavage by FEN1 are impaired when an 8-oxoG lesion is in the −1 position to a reduced AP site [28], showing that long patch BER, as well as short patch BER, may be compromised by DNA clustered damaged sites. The final stage of BER, ligation, is also sensitive to additional proximal lesions. The ligase III/XRCC1 complex seals the nick left after short patch BER whilst the nick remaining following long patch BER is sealed by ligase 1. Both of these proteins have been shown to have reduced activity of up to 3.3 fold when a base lesion is on the opposing DNA strand to the nick [27,28,32,33,40]. Indeed when two 8-oxoG lesions are positioned one and five bases in the positive orientation from a nick resulting from the repair of an AP site, ligation of the nick does not occur [38].

**Fig. 2:**
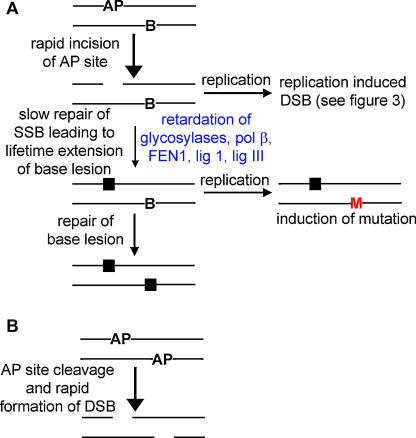
Schematic to summarise the processing of non-DSB clustered DNA damage. (A) A SSB, induced either directly from ionizing radiation or from cleavage of an AP site, inhibits the repair of an opposing base lesion until it itself has been repaired, thus limiting the formation of DSB. The opposing base lesion, however, impairs the repair of the SSB thus the lifetime of both lesions is increased. If the SSB encounters a replication fork then replication induced DSB could be formed. Increasing the lifetime of the base lesion increases the likelihood the base lesion will be unrepaired at replication and increases the chances of mutation induction. AP, abasic site; B, base lesion; M, mutated base; filled square, incorporated base. (B) Two opposing AP sites are rapidly cleaved to produce a DSB (unless the AP sites are within two bases in the positive direction).

Bistranded clustered damaged sites have the potential to give rise to DSB, if both lesions within the cluster are excised prior to repair of one of the lesions. Indeed this is the case for two AP sites where both AP sites are rapidly incised if they are in the negative orientation to each other or if they are separated by more than three bases in the positive orientation [31,38,41,42] (Fig. 2). If the AP sites are less than three bases apart in the positive orientation then incision of one AP site confers an inhibition of incision on the second AP site and DSB are slow to form. AP site clusters resistant to cleavage have also been seen in human haemopoetic cells following γ-irradiation [23]. There is, however, a hierarchy of lesion processing when base lesions are within the clustered damaged site. As previously discussed an AP site or SSB strongly impairs the excision of a base lesion so that a base lesion close to an AP site or SSB will not be excised until the AP site/SSB has been repaired, thus limiting the formation of DSBs. This has been clearly demonstrated when a SSB is opposing an 8-oxoG lesion in close proximity, where it was shown in nuclear extracts that there is a lag phase of 8-oxoG excision until some of the SSB has been repaired, and then 8-oxoG is excised [27] (Fig. 2). This hierarchy of repair was also seen in a more complex clustered damaged site comprising of a SSB and four different base lesions including an 8-oxoG lesion on the strand opposing the SSB and a 5-hydroxyuracil (hU) lesion on the same strand as the SSB. In this case the hU is excised before 8-oxoG leading to local denaturing of the nucleotides between the hU and the SSB, resulting in 8-oxoG lying on a region of single-stranded DNA. Thus 8-oxoG cannot be excised but formation of a DSB is avoided. It is predicted in this scenario that mutations would arise from the clustered damaged site [40]. If the sites of the 8-oxoG and hU lesions were exchanged, then the hU lesion is still excised before 8-oxoG resulting in formation of a DSB [43].

So far only bistranded clustered DNA damaged sites have been discussed, however clustered DNA lesions can also occur in tandem, whereby two or more lesions are in close proximity on the same DNA strand. Like bistranded clusters, repair of lesions within tandem clustered sites is also compromised. The presence of an 8-oxoG lesion three bases 5′ to another 8-oxoG lesion does not retard the repair of the first 8-oxoG, however if the 3′ 8-oxoG is excised first then OGG1 binding to the 5′ 8-oxoG lesion prevents repair synthesis by polymerase β to complete repair of the 3′ lesion [44]. Similarly, 8-oxoG has been shown to impair the repair of an AP site by up to 10 fold when positioned within five base pairs on the same DNA strand [45]. Both the incision of the AP site and the ligation of the resulting nick following repair synthesis are retarded by a nearby 8-oxoG lesion and when 8-oxoG is in position +1 the repair synthesis by polymerase β is also inhibited. Both the incision of the AP site and its subsequent rejoining are more greatly impaired by the presence of 8-oxoG in tandem than when 8-oxoG is on the opposite DNA strand [31,36,45]. Likewise 8-oxo-7,8-dihydroadenine (8-oxoA) may retard the incision of an AP site by APE1 up to 25 fold and by Fpg up to 45 fold [46]. In addition yeast, human and *E. coli* endonucleases III and VIII are impaired in their ability to completely remove tandem dihydrouracil (DHU) lesions positioned next to each other. The excision of the lesions is sequential and either lesion can be excised first. Yeast yNtg1p and *E. coli* Nth can only remove one DHU lesion, either the 3′ or 5′ lesion as the SSB resulting from excision of the first DHU lesion inhibits excision of the second DHU. Yeast yNtg2p cannot remove the 5′ DHU if the 3′ lesion is excised first but if the 5′ lesion is removed first then the 3′ DHU can be excised by yeast yNtg2p. The converse is true for human NTH and *E. coli* Nei, they can remove DHU remaining on the 3′ terminus of a 5′ cleaved fragment but not from a 5′ terminus of a 3′ cleaved fragment [47]. The excision of 8-oxoG by hOGG1 is reduced by 2 fold when a Tg lesion is positioned −1 to the 8-oxoG lesion but no impairment in 8-oxoG excision is seen when Tg is positioned +1 to the 8-oxoG lesion. There was also no reduction in Tg excision by Nth in either of the tested positions [48]. In addition primer extension on the strand opposing the 8-oxoG:Tg tandem lesion was reduced by both Klenow and yeast polymerase η when the lesions were in either orientation, indeed primer extension opposing the 5′-8-oxoG-Tg-3′ tandem lesion was almost blocked [48].

The preferred substrates of Fpg and OGG1 are the same, namely 8-oxoG and FapyG, and both glycosylases are classed as a bifunctional with an associated AP lyase activity. However, the AP lyase activity of OGG1 is very low so that the resulting AP site is generally cleaved by AP endonuclease. In contrast Fpg has a robust AP lyase activity. The mammalian protein NEIL1 removes 8-oxoG very poorly from double stranded DNA but can remove 8-oxoG present near single stranded DNA [49], whereas this activity has not been described for OGG1, Fpg or Nei. The differences in the activities of bacterial and eukaryotic BER proteins may influence the way these organisms process clustered DNA lesions, however further work needs to be undertaken to see if this is the case. Indeed, NEIL1 has been shown to play a role in the processing of complex DSB [50].

**Fig. 3:**
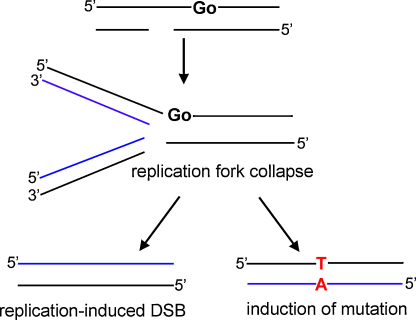
Schematic to show the induction of a replication induced DSB. If a cluster containing a SSB and a base lesion persists through to replication, both DSB and mutations may be induced. When the replication fork encounters the SSB it collapses, causing replication to stall (replicated strand shown in blue) with the ultimate result of a replication-induced DSB on one of the daughter chromosomes. Replication may bypass the 8-oxoG lesion by adding cytosine or mis-incorporating adenine opposite the 8-oxoG. For faithful repair, 8-oxoG would be removed from the template strand and replaced with guanine. However, if adenine has been wrongly inserted and 8-oxoG is removed before MutYH is able to correct the wrong nucleotide, base pairing will then follow the adenine leading to a fixed GC:TA transversion. Go, 8-oxoG.

As a consequence of the reduced repairability of clustered DNA damaged sites, the lifetime of the lesions within the clusters are increased relative to those of isolated lesions, as has been shown in cells [23,24]. If the clusters are not repaired, particularly in S- and G2 phase of the cell cycle, then the lesions in the cluster could encounter a replication fork thus replication induced DSB or mispairing of newly synthesised DNA across the lesions may occur. As a consequence of mispair, a mutation may eventually arise (Fig. 3).

## 3 *In vivo* assessment of cytotoxicity and/or mutability of clustered damage sites

Whilst the *in vitro* studies provide essential information on the challenge posed to individual enzymes within the BER pathway to process clustered damage, *in vivo* studies have given information on the cellular response to specifically designed clustered DNA damage as would be induced by ionizing radiation. Transformation of plasmids containing clustered damage has allowed the assessment of both the DSB formation and the mutation frequency of clustered DNA damage in *E. coli*.

It has been decidedly demonstrated *in vitro* that bistranded AP sites are incised with the ultimate result of DSB formation [36–38,42,51]. Bistranded AP sites and bistranded uracil residues result in rapid DSB formation within wild type *E. coli* [52–54], leading to the majority of studies focussing on the effect of uracils within a cluster. Extensive DSB formation is seen when two bistranded uracil residues are placed ≤seven base pairs apart [52,53] or tetrahydrofuran residues (an AP site analogue) ≥five bp apart [54] and transformed into *E. coli*, due to removal of both lesions to create two closely opposed SSBs. Consistent with this, if the cluster complexity is increased to contain two bistranded uracils with a vicinal 8-oxoG, extensive DSB formation is observed [38] irrespective of the location of 8-oxoG. Of the reduced number of plasmids able to undergo repair, deletions are the predominant mutation [52], with the mutation always occurring at the site of uracil rather than 8-oxoG in more complex clustered damage sites [38]. Tetrahydrofuran is unable to be cleaved by an AP lyase and therefore must be incised by an endonuclease. However, after transformation of bistranded tetraydrofurans into endonuclease deficient bacteria, DSBs are still formed [54]. The nucleotide excision repair pathway was not involved in the formation of the DSB, leading the authors to propose the existence of a back-up repair pathway. Such a back-up repair pathway may involve the lyase activity of bi-functional glycosylases. In addition, AP sites are highly toxic and can lead to collapse of replication forks which would prevent the plasmid from replicating and thus would appear as if DSB had been formed, as seen in *apn1*^−/−^, *apn2*^−/−^ yeast [55]. These are possible mechanisms for the conversion of bistranded AP sites to DSB and could also be possible mechanisms for the conversion of bistranded tetrahydrofurans to DSB in an endonuclease deficient background. The DSB formation of clustered damage sites after transformation into eukaryotic cells has also been assessed but is beyond the scope of this review.

To confirm that DSB formation observed from transformation of bistranded uracil residues is as a result of lesion processing and not as a result of the uracil residues persisting until replication, the clustered damaged site-containing plasmid was transformed into an ung1 deficient strain of *E. coli*. As ung1 is responsible for removal of uracil residues from DNA the uracil residues will not be excised via lesion processing and will therefore remain until replication. Following transformation of uracil-containing clustered damage sites into ung1 deficient *E. coli*, formation of DSB is not seen [38,52]. However, if AP site-containing plasmids are transformed into the same bacterial strain, extensive DSB formation is reported, comparable to that seen with wild type *E. coli*. The rapid formation of DSB resulting from a nick opposing an AP site implies that incision of AP sites is immediate, whereas DSB formation is spared when a base lesion opposes the nick, as the nick is repaired before excision of the modified base [56]. These results strongly suggest that uracil residues are removed and the resultant AP site is rapidly incised, thus DSBs are formed through abortive repair.

In contrast to clusters containing bistranded uracils or AP sites, DSB were not formed from clustered damage sites containing base lesions but an increased mutation induction was observed through lesion presence at replication, as a consequence of lifetime extension of the lesions within the cluster. An increase in mutation frequency is seen when plasmids containing 8-oxoG opposing another 8-oxoG lesion, a uracil residue, DHT or Tg are transformed into *E. coli*, compared to plasmids carrying a single isolated lesion. The mutations are almost always GC:TA transversions induced at the site of 8-oxoG and mutation frequency decreases as the inter-lesion separation increases [29,53,57–59]. These results demonstrate that lesion hierarchy is still evident. It was proposed that uracil, DHT or Tg is excised preferentially to 8-oxoG (probably due to the relative abundance of the relevant glycosylases within the cell) to produce a SSB, leading to an increased lifetime of 8-oxoG. As a consequence the 8-oxoG lesion is more likely to be present at replication and for a mutation to occur at this site. By using MutY and Fpg deficient *E. coli*, MutY (responsible for the removal of mis-incorporated adenine opposite 8-oxoG) was shown to be the predominant glycosylase to minimise mutation formation [53,59]. With all the bistranded clusters containing 8-oxoG studied in *E. coli*, the frequency of mutation at 8-oxoG is similar with ∼35–40% in MutY deficient bacteria [29,53,59].

Likewise, clusters containing two 8-oxoG lesions in tandem to each other, opposing a uracil residue did not result in formation of DSB but the mutation frequency at the site of 8-oxoG was increased over that of a single lesion [38]. Transformation of plasmid containing this cluster into MutY *E. coli* increased the mutation frequency of 8-oxoG-containing clusters ∼10-fold over single lesions and highlighted the sequential processing of these clustered damage sites. Following repair of the uracil residue the more 5′ of the two 8-oxoG lesions was preferentially excised leading to a higher induction of mutation at the more 3′ lesion [38].

Bistranded U/Tg clusters form extensive DSB in *E. coli* and of the few plasmids that are able to repair, small deletions within the immediate cluster site are seen [29]. If the cluster contains a nick opposing a uracil or an AP site then DSB are formed immediately, whereas an 8-oxoG or DHT placed in a cluster opposing a nick inhibits DSB formation [56]. The most mutagenic cluster investigated so far is nick/DHT which demonstrates a 5-fold increase in mutation frequency, compared to a 3.4-fold increase with an 8-oxoG/DHT cluster. It was determined that the direction of replication is not a factor in mutation induction.

When plasmids containing a tandem AP site and 8-oxoG lesion are transformed into *E. coli*, the mutation frequency is elevated [45]. The highest incidence of mutation is seen when the 8-oxoG is three bases 3′ to the AP site, an 11-fold increase over that of a single lesion. The mutation frequency of tandem 8-oxoG/AP site clusters was 2–3 fold lower than bistranded 8-oxoG/AP site clusters [53], possibly reflecting the loss of the lesion containing strand due to the persistence of the AP site. Unlike bistranded clusters containing 8-oxoG, the mutations were not restricted to GC:TA transversions at the site of 8-oxoG but were more varied. In contrast, no increase in mutation frequency is seen at the site of 8-oxoG when an 8-oxoG lesion is located immediately 5′ to the AP site. Parallel *in vitro* studies showed no base addition at the break site created from incision of the AP site within the 5′-8-oxoG-AP cluster [45] so the lack of mutation induction could be attributed to loss of the lesion-containing strand at replication, due to a persistent SSB, although this SSB could lead to a replication induced DSB.

The major findings on the *in vivo* effects of clustered damage sites are summarised in Table 1. Bistranded AP/AP, AP/nick, AP/uracil clusters are cytotoxic due to rapid incision of the AP site(s) resulting in DSBs. The major consequence of bistranded 8-oxoG/8-oxoG, 8-oxoG/DHT, 8-oxo/Tg, 8-oxoG/AP and 8-oxoG/uracil clusters is mutation induction, predominately GC:TA transversions, highlighting the importance of the abundance of different glycosylases in the cell. Formation of replication-induced DSBs are evident resulting from processing of 8-oxoG/AP clusters through the persistence of the AP site or resulting SSB at replication [60].

## 4 Concluding remarks

**Fig. 4:**
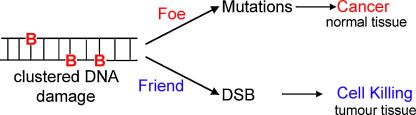
Ionizing radiation can be thought of as a “friend or foe”. If normal tissue is exposed to low doses of radiation (foe) mutations could be induced leading to genetic instability and tumourigenesis. However exposure of tumour tissue to high doses of radiation (friend) can kill the tumour cells. B, base lesion.

Based on the knowledge accumulating on non-DSB clustered DNA damage, the types of lesions in the cluster influence the efficiency of processing of the clusters which may be considered as friends or foes to humans, depending if formed in tumour cells or normal cells respectively (see Fig. 4).

A major application of ionizing radiation in medicine is in the treatment of tumours whereby the radiation is targeted to the tumour as best as possible and the tumour tissue is thought to be killed mainly by those DSB which have not been repaired and as a consequence are the most detrimental lesion produced by ionzing radiation. The number of DSB induced (∼10–20/Gray) by γ-radiation [61–63] and the speed at which tumour cells divide make ionizing radiation very effective for the treatment of tumours. Processing of the lesions within non-DSB clustered DNA damage induced by ionizing radiation can lead to the formation of additional DSB in both prokaryotic [22] and eukaryotic [64] cells. In addition delayed repair of SSB within radiation-induced non-DSB clustered damaged sites can result in replication induced DSB [60]. The induction of replication DSB in addition to the prompt DSB induced by ionizing radiation adds to the effectiveness of ionizing radiation as a therapeutic tool particularly as prompt DSB are repaired mainly by non-homologous end joining whereas replication-induced DSB require homologous recombination [65,66]. Therefore targeting proteins of the base excision repair pathway may be an approach to increase the efficacy of ionizing radiation since SSB are present in some clustered damage sites, either directly formed by ionizing radiation or by processing of base lesions. If the SSB cannot be repaired or the repair is delayed, such that SSB meet replication forks, a replication induced DSB results [67]. Since clustered DNA damaged sites make greater use of the LP-BER pathway than isolated lesions [27,45,68] and that ligase III/XRCC1 complex cannot substitute for ligase 1 to complete LP-BER [69], the proteins involved in LP-BER may be potential candidates to target. Therefore by inhibition of FEN1 or ligase 1 as examples, it may be possible to increase the cytotoxicity of cells to radiation. Indeed one study has shown that treating cancerous cells with sub-lethal doses of ligase 1 inhibitors does increases their radiosensitivity due to the higher expression of ligase 1 compared to normal cells [70]. In addition inhibition of FEN1, the protein responsible for the removal of the DNA flap following strand displacement during LP-BER, has been shown to block LP-BER and enhance the cytotoxic effect of temozolomide in colon cancer cells [71]. Inhibiting FEN1 may also increase the cytotoxic effect of ionizing radiation.

Additionally, if a replication induced DSB is produced from a clustered damage site, and in particular a more complex cluster and is subsequently repaired by homologous recombination, then this ‘repair’ may lead to formation of a *de novo* clustered damage site which would then require further processing.

However not all exposure to ionizing radiation is beneficial and the induction and processing of non-DSB clustered damage can be thought of as a double edged sword when considering radiation doses received environmentally, through diagnostic radiology and through exposure of normal tissue during radiotherapy. Whilst at high exposure this property of ionizing radiation can help to kill tumours, probably via apoptosis, at low exposures to normal tissue, the induction and processing of non-DSB clusters can be detrimental. It has often been postulated that the most serious lesion arising as a result of ionizing radiation exposure is DSB, as the role of a DSB in inducing chromosomal translocations, mutations and cell death is well established. However at lower environmental levels of radiation exposure (1–5 mGy) one prompt DSB is induced on average per 20–100 cells. Based on the 4–8 times higher yields of non-DSB clustered DNA damage/Gy as compared with that of DSB/Gy [19,20] it is postulated that one non-DSB clustered DNA damage is induced on average per 5–20 cells. Therefore some cells will only have non-DSB clusters present, hence there is a probability of inducing a mutation [2]. Indeed human monocytes repair clustered lesions poorly, leading to the conversion of bistranded clusters to lesions that affect only one DNA strand at replication. Although repair of a single lesion in one of the daughter strands will be more efficient than that of a cluster, replication of lesion containing DNA is mainly via translational synthesis [72] and is error prone, thus there is a potential for mutation induction [23,73]. As a consequence the induction of clustered damaged sites is thought to contribute to the detrimental consequences of ionizing radiation such as carcinogenesis. Indeed, 10–15% of all lung cancer deaths can be attributed to the exposure of radon [74], a natural source of high LET ionizing radiation. Similarly during radiotherapy, when a tumour is targeted with ionizing radiation the normal tissue surrounding the tumour also receives a lower dose of radiation than that to the tumour. In this case a normal cell may receive a single DSB, which has a high probability of being repaired with high efficiency for sparsely ionizing radiation but also non-DSB clusters may be induced at levels 4× that of prompt DSB [19,20]. The delayed repair of non-DSB clustered damage if present at replication, may lead to the induction of mutations through mis-incorporation of bases, the formation of replication induced DSB and ultimately chromosomal aberrations, genetic instability and tumourigenesis.

## Conflict of interest statement

The authors declare there are no conflicts of interest.

## Figures and Tables

**Table 1:** Summary of results following transformation of non-DSB clustered-lesion containing plasmids into *E. coli*. na: not assessed.

Lesions within the cluster	Cytotoxic or mutagenic	Predominant type of mutation
U/U	Cytotoxic	Deletion
U/U and 8-oxoG	Cytotoxic	Deletion at U
AP/AP	Cytotoxic	Deletion
Nick/U	Cytotoxic	na
Nick/AP	Cytotoxic	na

U/Tg	Cytotoxic	Deletions at Tg

U/8-oxoG	Mutagenic	GC:TA transversion at 8-oxoG
U/8-oxoG and 8-oxoG	Mutagenic	GC:TA transversion at 3′ 8-oxoG

8-OxoG/8-oxoG	Mutagenic	GC:TA transversion
8-OxoG/DHT	Mutagenic	GC:TA transversion at 8-oxoG
8-OxoG/Tg	Mutagenic	GC:TA transversion at 8-oxoG

Tandem AP–8-oxoG	Mutagenic	GC:TA transversions at 8-oxoG
